# Mutations in GFAP Disrupt the Distribution and Function of Organelles in Human Astrocytes

**DOI:** 10.1016/j.celrep.2018.09.083

**Published:** 2018-10-23

**Authors:** Jeffrey R. Jones, Linghai Kong, Michael G. Hanna, Brianna Hoffman, Robert Krencik, Robert Bradley, Tracy Hagemann, Jeea Choi, Matthew Doers, Marina Dubovis, Mohammad Amin Sherafat, Anita Bhattacharyya, Christina Kendziorski, Anjon Audhya, Albee Messing, Su-Chun Zhang

**Affiliations:** 1 Waisman Center, University of Wisconsin-Madison, Madison, WI 53705, USA; 2 Molecular and Cellular Pharmacology Training Program, University of Wisconsin-Madison, Madison, WI 53705, USA; 3 Department of Neuroscience, School of Medicine and Public Health, University of Wisconsin-Madison, Madison, WI 53705, USA; 4 Department of Neurology, School of Medicine and Public Health, University of Wisconsin-Madison, Madison, WI 53705, USA; 5 Department of Biomolecular Chemistry, University of Wisconsin-Madison, Madison, WI 53706, USA; 6 Department of Biostatistics and Medical Informatics, University of Wisconsin-Madison, Madison, WI 53706, USA; 7 Department of Veterinary Medicine, University of Wisconsin-Madison, Madison, WI 53706, USA; 8 Program in Neuroscience & Behavioral Disorders, Duke-NUS Medical School, Singapore, Singapore; 9 Lead Contact

## Abstract

How mutations in glial fibrillary acidic protein (GFAP) cause Alexander disease (AxD) remains elusive. We generated iPSCs from two AxD patients and corrected the GFAP mutations to examine the effects of mutant GFAP on human astrocytes. AxD astrocytes displayed GFAP aggregates, recapitulating the pathological hallmark of AxD. RNA sequencing implicated the endoplasmic reticulum, vesicle regulation, and cellular metabolism. Corroborating this analysis, we observed enlarged and heterogeneous morphology coupled with perinuclear localization of endoplasmic reticulum and lysosomes in AxD astrocytes. Functionally, AxD astrocytes showed impaired extracellular ATP release, which is responsible for attenuated calcium wave propagation. These results reveal that AxD-causing mutations in GFAP disrupt intracellular vesicle regulation and impair astrocyte secretion, resulting in astrocyte dysfunction and AxD pathogenesis.

## In Brief

Jones et al. study the structure function relationship of GFAP on astrocytes using Alexander disease patient-derived induced pluripotent stem cells. Mutations in GFAP result in mislocalization of organelles and functional consequences such as reduced ATP release and attenuated calcium wave propagation. Genetic correction of mutant GFAP rescues these defects.

## Graphical Abstract

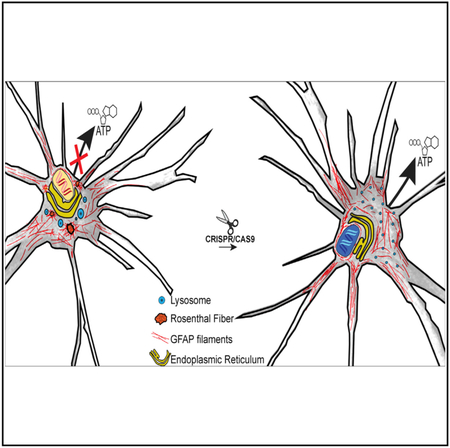

## INTRODUCTION

Astrocytes account for approximately 20%–40% of the cells in the human brain (Sun et al., 2017; Zhang et al., 2016). Astrocytes are integral components of a functioning brain, essential for metabolic support (Bak et al., 2006; Schurr and Payne, 2007; Shank et al., 1985), ion homeostasis (Bellot-Saez et al., 2017; D’Ambrosio and Gordon, 2002; Hertz and Chen, 2016), synapse regulation, and the coupling of neuronal metabolism and waste disposal to the blood supply (Iliff et al., 2012). Aside from active crosstalk at the synapse, astrocytes interact with neighboring glial cells and vasculature to participate in myelination and blood-brain barrier (BBB) formation (Gard et al., 1995; Ishibashi et al.,2006; Lippmann et al., 2011). These functions are mediated by secretion of glio-transmitters, trophic factors, and cytokines (Bhat and Pfeiffer, 1986; Domingues et al., 2016; Gard et al., 1995; Tanuma et al., 2006; Verkhratsky et al., 2016), which is, at least in part, coordinated through intercellular astrocyte signaling in the form of calcium (Ca^2+^) waves (Kreft et al., 2004; Ramamoorthy and Whim, 2008; Verkhratsky and Nedergaard, 2018; Zhang et al., 2007).

Intracellular astrocyte calcium signaling is triggered by multiple factors, including glutamate at the synaptic cleft, which stimulate G protein-coupled receptors on the membrane, leading to the production of IP3. IP3 activates IP3R2 receptors on the endoplasmic reticulum (ER), resulting in intracellular calcium release (Golovina and Blaustein, 2000; Scemes and Giaume, 2006; Sheppard et al., 1997) and subsequent exocytosis (Coco et al., 2003; Mothet et al., 2005; Pascual et al., 2005), including the release of ATP, which in turn propagates intercellular calcium waves (Anderson et al., 2004; Coco et al., 2003; Cornell-Bell et al., 1990). Astrocytic calcium signaling and secretion is tightly regulated to enable proper astrocyte function and maintenance of CNS homeostasis. How this signaling is regulated and what functional consequence result from disrupted signaling in astrocytes are not known.

Mutations in the astrocyte intermediate filament glial fibrillary acidic protein (GFAP) result in Alexander disease (AxD) (Brenner et al., 2001). AxD is a progressive and fatal neurological disorder characterized by astrocytic cytoplasmic inclusions containing GFAP, termed Rosenthal fibers (RFs). Disease presentation is associated with multiple phenotypes, including myelination abnormalities, gait ataxia, megalencephaly, and susceptibility to seizures (Brenner et al., 2009; Messing et al., 2012; Prust et al., 2011). The mechanism by which GFAP mutations lead to astrocyte dysfunction and global neurological changes is unknown.

Transgenic animals with orthologous AxD-causing mutations recapitulate RF pathology and exhibit age-dependent changes in RF morphology and distribution, similar to observations made in patients (Hagemann et al., 2006; Sosunov et al., 2017). These mice are susceptible to kainic acid-induced seizures but do not exhibit gross neurological changes. Interestingly, overexpression of human wild-type GFAP is sufficient to produce RFs and exacerbates orthologous murine GFAP mutations (Hagemann et al., 2006; Messing et al., 1998), suggesting a potential toxic gain of function. Conversely, GFAP-null mice are viable and exhibit no gross anatomical changes, though they exhibit vascular defects (Liedtke et al., 1996, 1998; Pekny et al., 1998), along with changes in long-term potentiation (LTP) and long-term depression (LTD) (McCall et al., 1996; Shibuki et al., 1996), hinting a possible loss of function. The mild phenotypes in transgenic rodents relative to patients may be associated with species difference. Human astrocytes are much larger with more processes, contact 100 times more synapses, and propagate calcium waves 5 times faster than rodents (Navarrete et al., 2013; Sun et al., 2013). Hence, the ability to analyze the effect of mutant GFAP directly on human astrocytes will complement the existing models and help produce insights into the pathogenesis of AxD.

We generated induced pluripotent stem cells (iPSCs) from two unrelated type II AxD patients and corrected the mutations using CRISPR/Cas9. We found that AxD iPSC-derived astrocytes exhibit GFAP aggregates, which were not apparent after genetic correction of GFAP mutations. Importantly, we found that AxD astrocytes displayed abnormal organelle morphology and distribution coupled with impaired calcium wave propagation through reduced ATP release.

## RESULTS

### Mutations in GFAP Do Not Alter Astrocyte Differentiation

**Figure 1.**
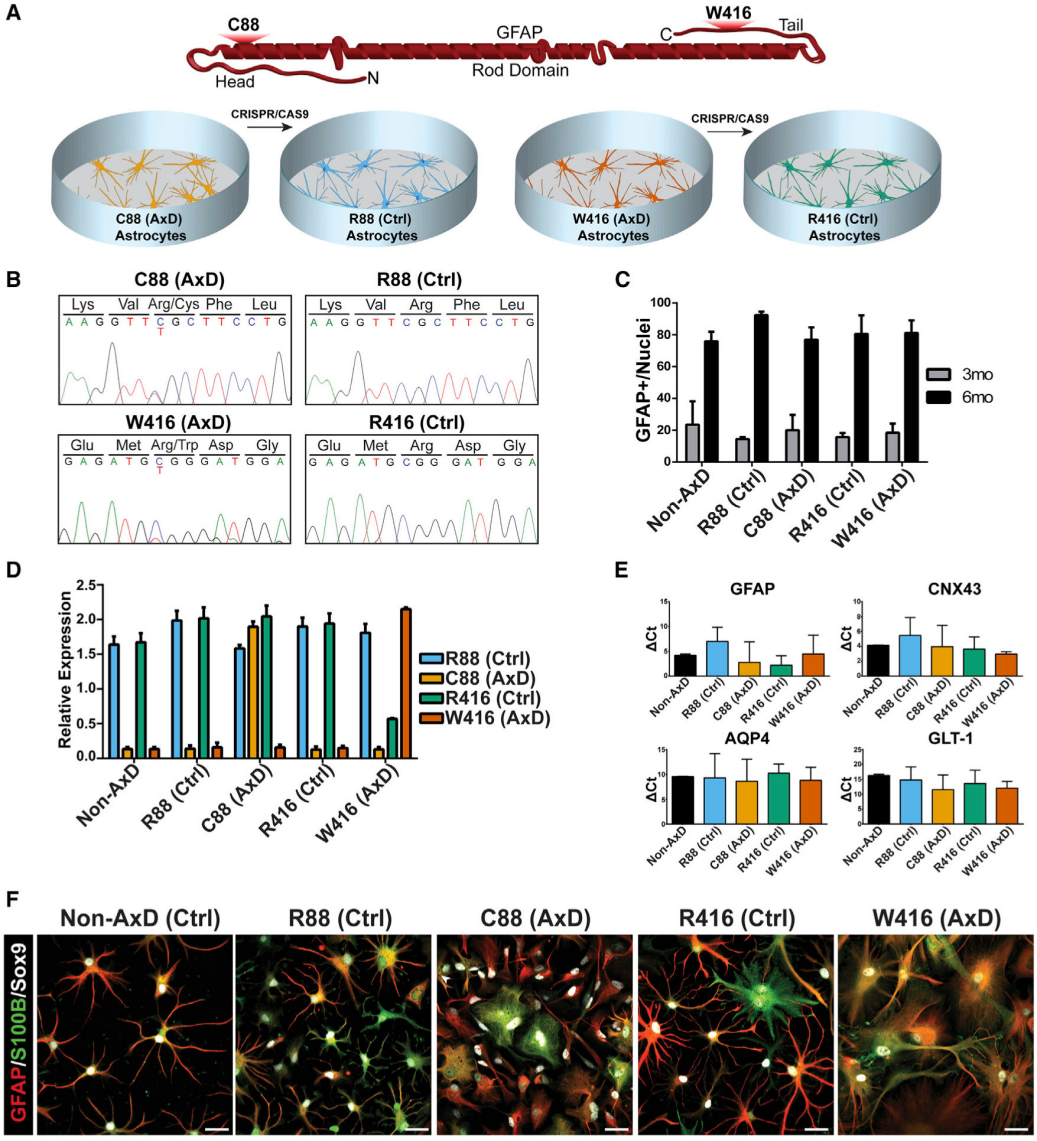
Effects of GFAP Mutations on Astrocyte Differentiation (A) Cartoon depicting relative locations of AxD mutations on a GFAP monomer along with an introduction of cell lines. (B) Sanger sequencing of patient-derived IPSCs and corrected isogenic lines. (C) Quantification of GFAP+ cells at 3 months (gray) and 6 months (black) during astrocyte differentiation (n = 4). (D) TaqMan SNP results showing relative RNA detection for each specific probe (n = 3). (E) Expression of astrocyte-associated markers by qPCR (n = 4). (F) Immunocytochemistry of 6 month astrocytes for GFAP, S100B, and Sox9. Scale bars, 100 μm. Data are represented as mean ± SD.

To investigate the effect of mutant GFAP in human astrocytes, we generated iPSCs from two unrelated, heterozygous AxD patients. A C→T transition at nucleotide position 262 results in an arginine-to-cysteine mutation at amino acid residue 88 (R→C88) in one patient, and a C→T transition at nucleotide position 1246 results in amino acid residue 416 switching from an arginine to a tryptophan (R→W416) in the other patient. The two mutations occur on opposite ends of the GFAP gene: C88 occurs in the first alpha helix within the rod domain, and W416 occurs within the tail domain (Figure 1A). Stable, embryonic stem cell-like colonies were apparent after reprogramming, and these colonies were immunopositive for a panel of pluripotency-associated markers, including OCT4, NANOG, SOX2, Tra-1-80, and Tra-1-60 (Figure S1A). For all experiments, two sub-clones of each iPSC cell line were analyzed. iPSCs were chromosomally stable after propagation on Matrigel for more than 40 passages (Figure S1C).

To control for intrinsic genetic background variation, we corrected the mutated allele in each patient iPSC line using CRISPR/Cas9 (Figures 1A and S1B). Generation of isogenic control cell lines also allowed analysis of similarities and differences between the two mutations. As with the mutant lines, two sub-clones of each isogenic line were used throughout the experiments. DNA sequencing analysis indicated that C88 and W416 (AxD mutant lines) were changed to R88 and R416 (corrected control lines), respectively (Figure 1B). The genetically modified iPSCs were expanded in the same culture environment for 40 passages and expressed OCT4, NANOG, SOX2, Tra-1-80, and Tra-1-60 (Figure S1A). All lines retained normal karyotypes (Figure S1C). Thus, we have generated isogenic pairs of iPSCs.

We first asked if GFAP mutations alter astrocyte differentiation. We differentiated both the disease and isogenic iPSCs to astrocytes alongside an unrelated, sex-matched embryonic stem cell line (H1; non-AxD) using our established protocol (Krencik et al., 2011). High-content immunocytochemical analysis indicated that approximately 20% of the cells were GFAP+ at 3 months of differentiation, which increased to 80% by 6 months (Figure 1C). To determine whether we were selecting against mutant GFAP-expressing cells during the lengthy differentiation process, we performed a SNP TaqMan assay on RNA collected from 6 month astrocytes. We found that both the normal and mutant RNA transcripts were detected at similar levels in mutant lines, whereas no mutant allele was detected in the isogenic or unrelated control cell lines (Figure 1D). Thus, mutant GFAP expression is retained in disease lines after 6 months of differentiation in culture.

After 6 months of differentiation, astrocyte-associated markers GFAP, CNX43 (*GJA1*), AQP4, and GLT-1 (*SLC1A2*) were detected via qPCR (Figure 1E), with no significant difference observed in transcript levels between disease and control lines. The astrocyte cultures did not express neuronal or oligodendrocyte markers via qPCR (Figure S1D). The GFAP+ astrocytes differentiated from all lines exhibited stellate morphology and co-expressed S100B and SOX9, as shown by immunocytochemistry (Figure 1F). Thus, both the mutant and their isogenic iPSCs give rise to astroglial cells at a similar efficiency.

### AxD Astrocytes Exhibit GFAP Aggregation

**Figure 2.**
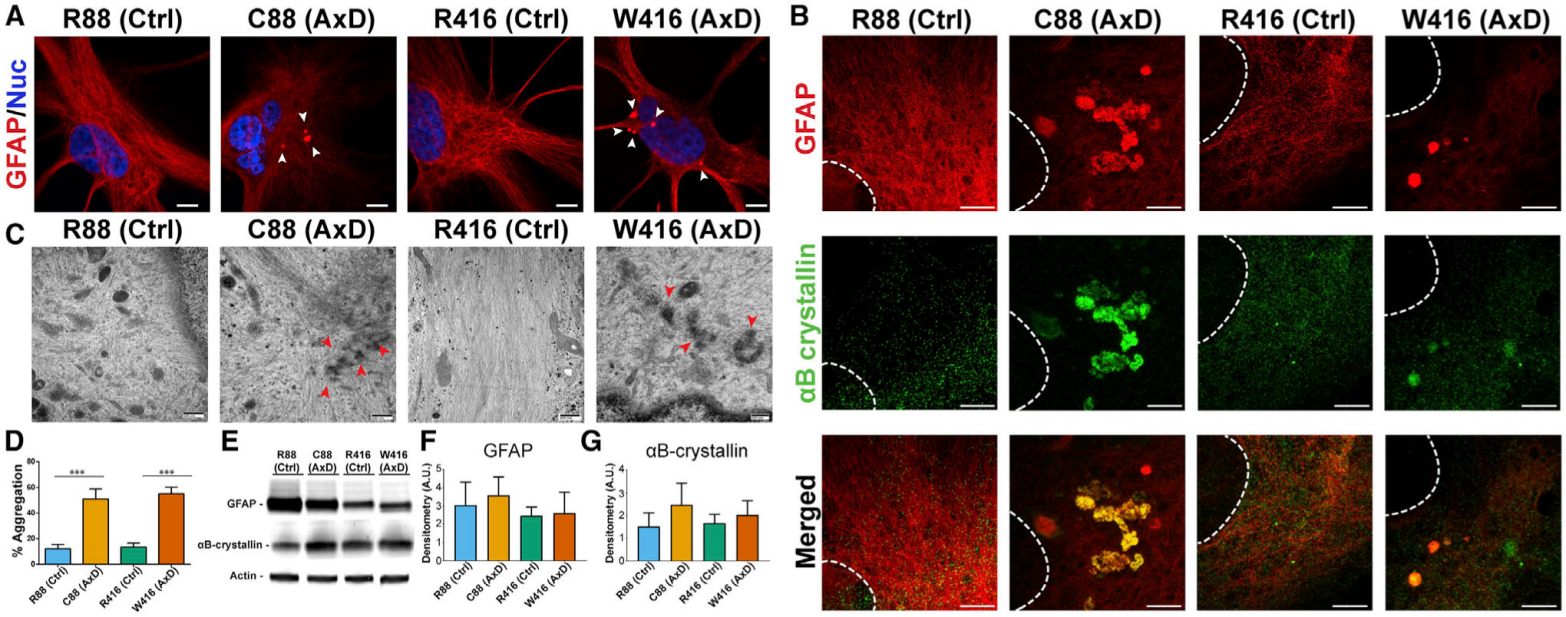
GFAP Expression and Aggregation in Differentiated Astrocytes (A) Immunocytochemistry for GFAP showing perinuclear GFAP+ puncta (arrowheads). Scale bar, 10 μm. (B) STED microscopy images on astrocytes stained for GFAP (red) and αB-crystallin (green). Scale bar, 2 μm. (C) Electron micrographs of aggregated GFAP (arrowheads). Scale bar, 800 nm. (D) Quantification of cells exhibiting punctate GFAP signal over total GFAP population (n = 4). (E) Western blot from 6 month astrocyte lysates probed for GFAP, xαB-crystallin, and actin. (F) Densitometry analysis for GFAP (n = 3). (G) Densitometry analysis for αB-crystallin (n = 3). *p ≤ 0.05. Data are represented as mean ± SD.

The pathological hallmark of AxD is the presence of proteinaceous inclusions of GFAP, termed RFs, in astrocytes. By immunostaining AxD and isogenic control astrocytes for GFAP, we found that control astrocytes exhibited filamentous GFAP throughout the cell body and major processes. In AxD astrocytes, we observed punctate GFAP inclusions in a perinuclear area (Figure 2A). RFs in AxD patients and transgenic mouse models contain the small heat shock protein αB-crystallin (Heaven et al., 2016; Iwaki et al., 1989). Super-resolution stimulated emission depletion (STED) imaging showed that αB-crystallin and vimentin co-localized with non-filamentous, punctate GFAP in both AxD mutant lines (Figures 2B and S2A). Under electron microscopy, we observed bundles of disorganized intermediate (GFAP) filaments in perinuclear areas of AxD astrocytes but not isogenic astrocytes (Figure 2C). RF localization appeared exclusively perinuclear in both disease lines, resembling early RFs observed in *in vivo* systems (Brenner et al., 2009; Sosunov et al., 2017). The size and shape of aggregates varied in disease-derived astrocytes, with less numerous but larger RFs (400 nm to 2.8 μm) observed in the C88 line and smaller (100–400 nm) but more numerous RFs in the W416 line. Quantification revealed that about 50% of astrocytes from AxD lines contained perinuclear, non-filamentous, punctate GFAP staining (Figure 2D).

Western blotting revealed that although the GFAP levels varied between individuals, the total protein levels for GFAP and αB-crystallin were similar between disease and isogenic controls (Figures 2E–2G). Hence, cultured AxD astrocytes exhibit characteristic RF-like pathology similar to those seen in AxD patients and animal models.

### Mutations in GFAP Alter ER, Vesicle Regulation, and Cellular Metabolism Transcripts

**Figure 3.**
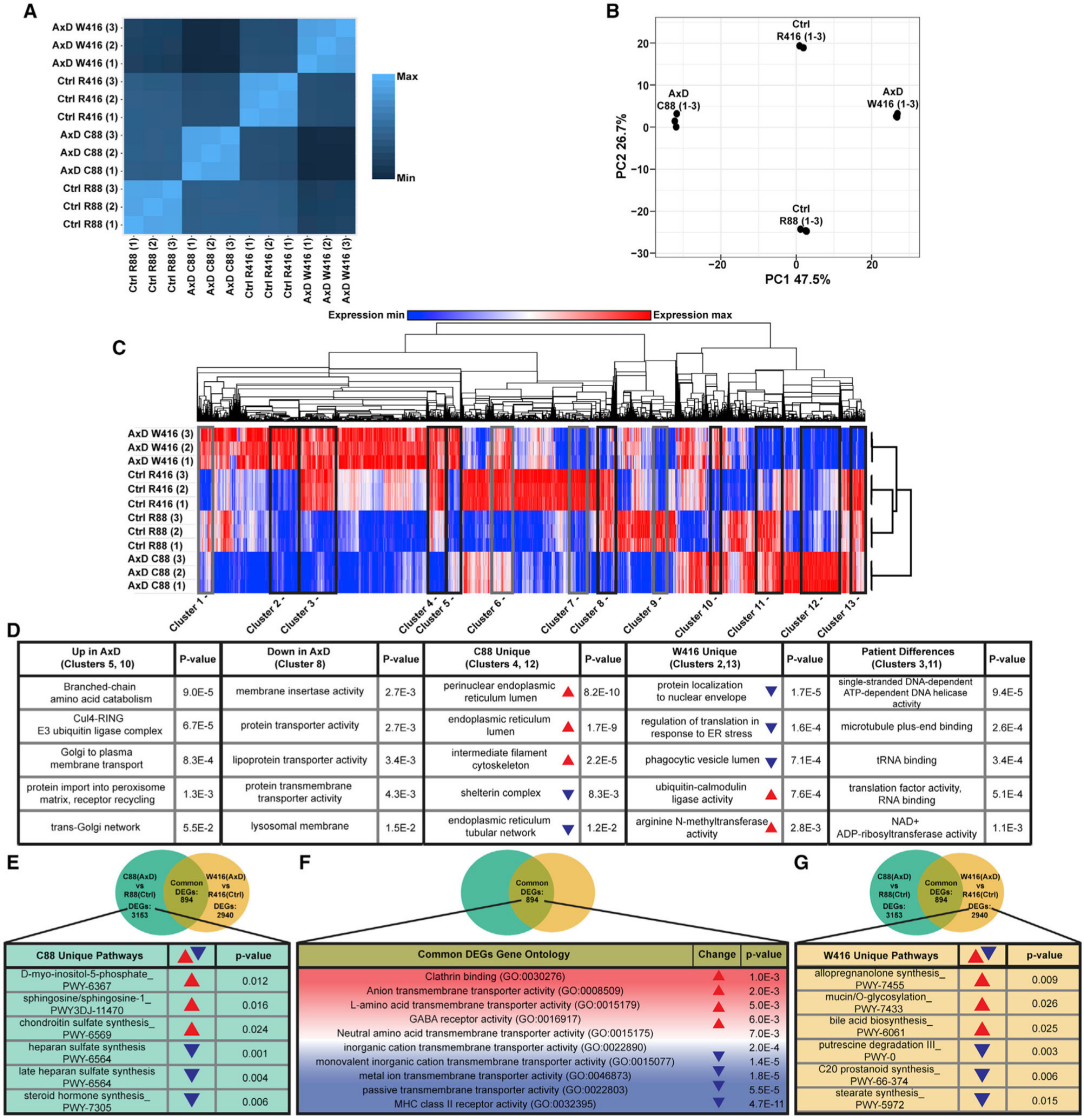
RNA-Seq and Pathway Analysis (A) Spearman’s correlation heatmap of total TPM values. Lighter color indicates higher similarities. (B) Principal-component analysis of top 1,000 most variable transcripts. (C) Hierarchical clustering heatmap of full transcript dataset. For gray boxed clusters, see Figure S3. (D) Gene Ontology analysis of transcripts comprising clusters of interest. Red arrowheads indicate upregulation and blue arrowheads represent downregulation. Ontologies were sorted by significance as determined by p value. (E) DEG analysis of full transcript dataset followed by analysis to determine unique pathways for each mutation. Table shows Gene Ontology analysis on transcripts uniquely changed in C88 (AxD). (F) DEGs that were common between AxD mutant lines and subsequent pathway analysis by Gene Ontology. (G) DEG analysis of full transcript dataset followed by analysis to determine unique pathways for each mutation. Table shows Gene Ontology analysis on transcripts uniquely changed in W416 (AxD). Red arrowheads indicate upregulation and blue arrowheads represent downregulation.

To elucidate potential pathways altered as a consequence of GFAP mutations in astrocytes, we performed RNA sequencing on disease and corrected astrocytes (Data S1 and S2). Spearman’s correlation of total transcriptomes showed divergent transcriptomes between the two individuals, even after genetic correction (Figure 3A), which highlights the individual variation. Interestingly, there was a lower correlation between the two disease groups (C88 and W416) than between the two control lines or between the control and disease lines. This result indicates that although there is expected genotypic variation between the individuals from which the lines were derived, there is a larger variation in gene expression caused by the individual mutations in GFAP.

To compare the contribution of transcriptional changes associated with patient genetics or GFAP mutations, we performed a principal-component analysis (PCA) of the top 1,000 most variable transcripts across all samples (Figure 3B). Corrected lines separated only by PC2 (26.7% of variance) and disease lines separated only by PC1 (47.5% of variance). We then correlated the top 1,000 most variable transcripts back to the full transcriptome dataset via an unbiased circle of correlation and observed that both corrected lines were more alike than the two mutant lines (Figure S3A), further supporting our interpretation of differential transcriptional responses to mutated GFAP. Thus, PCA and Spearman’s correlation demonstrate that the most variable transcripts were likely not contributed by genetic background but rather differential transcriptional responses to GFAP mutations.

To understand the consequence of the differential transcriptional responses to different GFAP mutations, we performed hierarchical clustering on the full transcriptomic dataset (Figures 3C and S3B). Clustering via transcripts (Data S3) highlighted regions in which transcriptional changes were similar in both mutant lines (Figure 3D, clusters 5, 8, and 10), mutation-specific (Figure 3D, clusters 2, 4, 12, and 13), or genotypic patient differences (Figure 3D, clusters 3 and 11). Gene Ontology analysis of clusters associated with mutant GFAP demonstrated similarities, regardless of the direction of transcriptional change. Pathways commonly changed in both mutant lines suggest changes in the regulation of membrane and protein transport (Figure 3D, clusters 5, 8, and 10). Even pathways unique to each mutant were consistent. These ontologies included membrane regulation, ER regulation, and protein trafficking along with endosome and lysosome regulation (Figure 3D, clusters 2, 4, 12, and 13). We also analyzed clusters in which corrected controls were outliers (Figures S3B and S3C) observing ontologies associated with general transcription, cell cycle, immune related, and trafficking (Figure S3C). Thus, despite unique transcriptional responses to GFAP mutations in each disease line, the predicted affected pathways were similar in both mutant lines.

To determine unique and common differentially expressed genes (DEGs) in an unbiased fashion, each disease line was compared with its respective control. Each mutant had approximately 3,000 unique DEGs that were ≥ 2-fold up- or downregulated (Data S4). Eight hundred ninety-four DEGs were commonly changed between both mutants. Multiple pathway analyses were performed on unique and common DEGs (both up- and downregulated), as well as on non-DEGs (genes changed <2-fold between mutant and disease lines) (Figures 3E–3G). These analyses further supported our PCA and clustering analysis, which indicated alteration in membrane-related proteins and specified terms, including clathrin binding, receptor and transporter activity, vesicular formation and trafficking, and protein degradation (Figure 3F), while giving us specific target transcripts.

Thus, our multiple RNA sequencing analyses demonstrates that different disease causing mutations in GFAP result in divergent transcriptional changes with predicted overlapping functions. Though individual pathways are predicted depending on algorithm used, we commonly observed pathways that broadly implicate the ER, vesicle regulation, and cellular metabolism, and we therefore investigated these pathways further.

### AxD Astrocytes Exhibit Altered ER and Lysosome Structures

**Figure 4.**
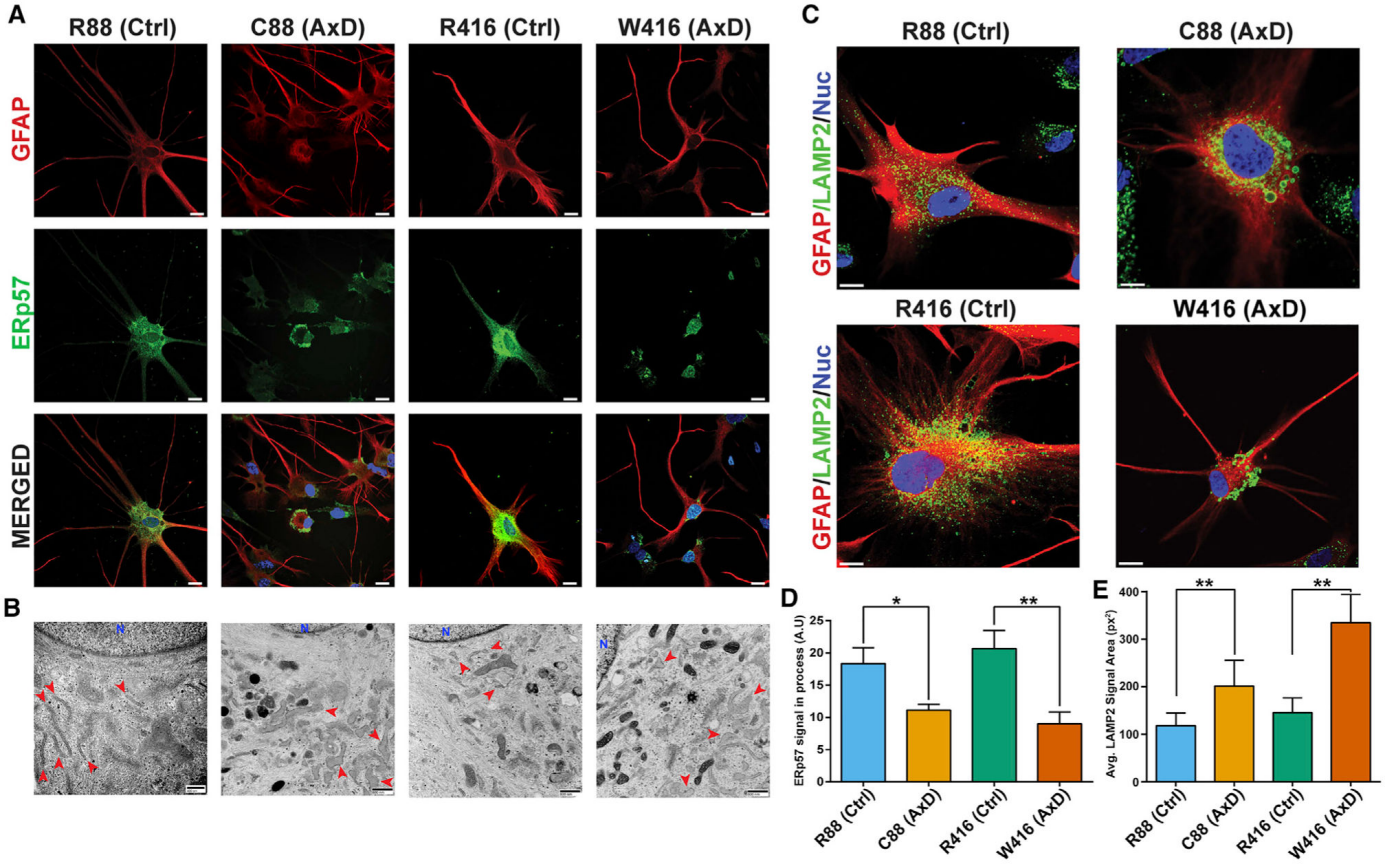
Distribution and Morphology of ER and Lysosomes (A) Panel of immunofluorescent images of 6 month astrocytes stained for GFAP (red) and the ER marker ERp57 (green). Scale bars, 50 μm. (B) Electron micrographs comparing morphology of ER (red arrows) between groups. Scale bars, 800 nm. (C) Immunostaining of lysosomes via LAMP2 (green) on 6 month astrocytes. Scale bars, 10 μm. (D) Average signal of ERp57 in astrocyte processes between groups (n = 3). (E) Average LAMP2 signal area between groups. *p ≤ 0.05 and **p = 0.001–0.01 Data are represented as mean ± SD.

RNA sequencing analysis indicated disease-specific changes in transcripts associated with the ER, vesicle regulation, protein degradation, and metabolism. Immunostaining for the ER protein ERp57 demonstrated significantly more somatic, non-reticular ER in both the disease lines (Figures 4A and 4D). In contrast, isogenic and normal astrocytes exhibited ERp57 signal throughout the cell (Figure 4A). Transmission electron microscopy analysis confirmed a swollen and largely non-reticular ER in disease astrocytes (Figure 4B) but not controls. Immunofluorescent visualization of other cytoskeletal components such as actin and microtubules did not show any difference between AxD and non-AxD cells (Figures S2C and S2D), suggesting that ER redistribution is not caused by loss of cytoskeleton structure and therefore loss of microtubule mediated ER trafficking.

RNA sequencing pathway analysis indicated trafficking pathways, specifically clathrin pathways, as the most upregulated common to both mutations (Figure 3F). Clathrin is involved in endo- and exocytosis and the generation of vesicles such as lysosomes (Coutinho et al., 2012; Luzio et al., 2014). Immunostaining for clathrin and quantification of puncta by high-content analysis yielded no significant difference between isogenic controls and disease astrocytes (Figure S4A). Analysis of basal endocytosis rates on live astrocytes was performed via fluorescent dextran uptake assay and yielded no significant differences between any of the lines (Figure S4B).

Besides endocytosis, clathrin plays important roles in lysosome trafficking and exocytosis (Jaiswal et al., 2002; Sreetama et al., 2016; Zhang et al., 2007). Immunostaining for the lysosomal marker LAMP2 demonstrated that these vesicles varied in both size and distribution in AxD cells relative to the uniform size in controls (Figure 4C). The vesicles were on average significantly larger and more concentrated in the perinuclear area compared with processes in the AxD astrocytes (Figure 4E). Interestingly, GFAP+ inclusions and LAMP2+ vesicles did not regularly co-localize, and no difference was observed in the Golgi apparatus between cell lines (Figures S4C and S4D). These data suggest a functional role of the GFAP protein in ER and lysosome morphology, distribution, and function.

### AxD Astrocytes Display Deficits in Propagating Calcium Waves

**Figure 5.**
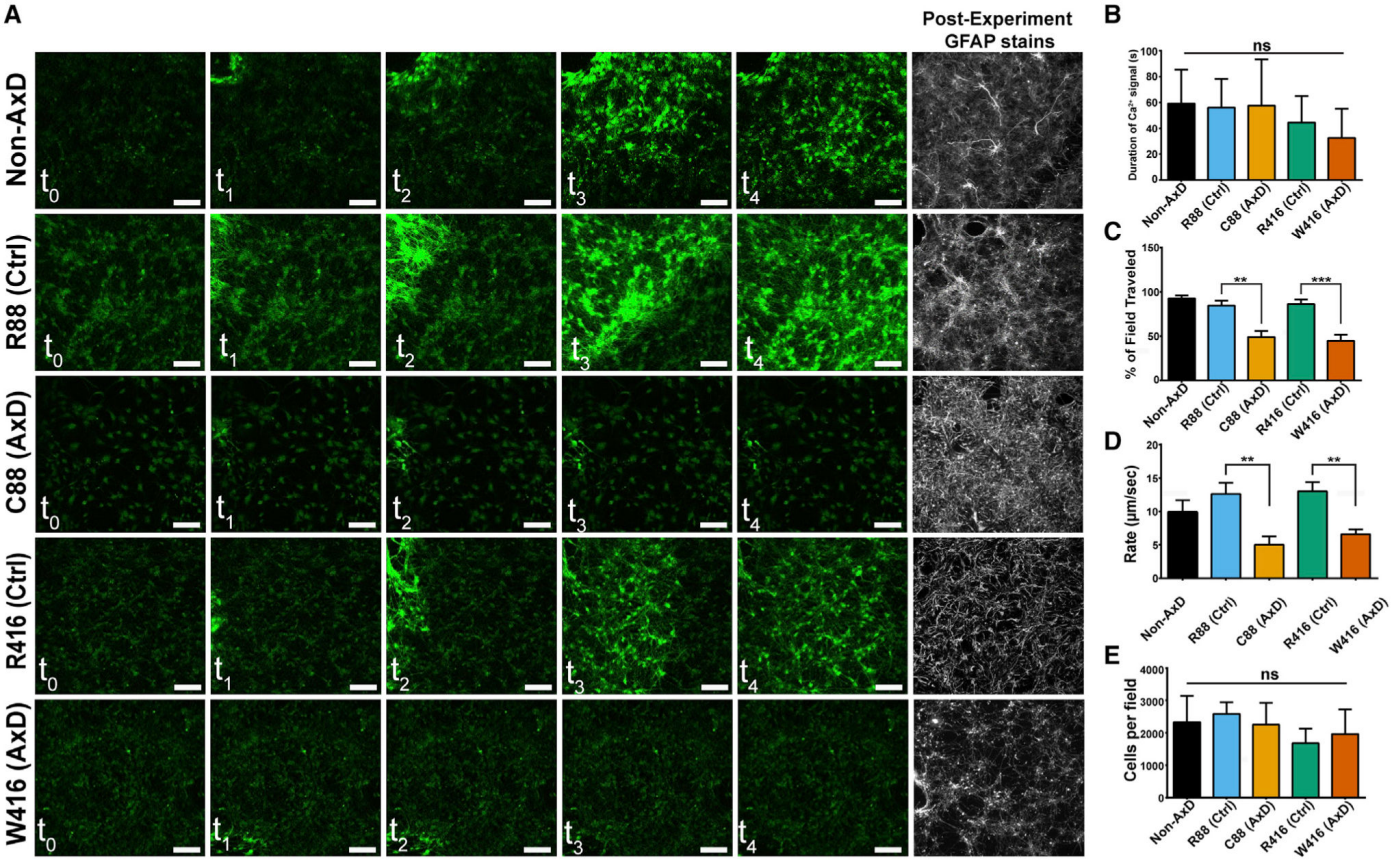
Mechanical Stimulation-Induced Calcium Waves (A) Still images over the duration (2 min) of mechanical stimulation with accompanying post-experiment GFAP stains. (B) Average duration of increased calcium signal above threshold ± SD (n = 10). (C) Average percentage of field traveled by Ca^2+^ wave (n = 10). (D) Rate of Ca^2+^ wave propagation (n = 10). (E) Post-experiment quantification of cells per field (n = 10). **p = 0.001–0.01 and ***p < 0.001. Scale bars, 100 mμm. Data are represented as ± SEM unless otherwise noted.

Astrocyte-to-astrocyte communication and gliotransmission, in part, rely on the propagation of calcium waves and the ability to secrete molecules. These processes depend on proper ER and, to some extent, lysosome function (Jaiswal et al., 2002; Parekh and Putney, 2005; Zhang et al., 2007). The altered distribution and morphology of ER and lysosomes (Figure 4) suggest potential biological consequences. When mechanically stimulated, AxD astrocytes exhibited attenuated ability to propagate calcium waves over time compared with controls (Figure 5A). Cells adjacent to the stimulation site increased in fluorescent intensity for similar amounts of time as controls (Figure 5B), suggesting that the astrocytes respond to the mechanical stimulation by releasing intracellular stores of Ca^2+^. However, the AxD astrocytes failed to propagate waves across the observable field, whereas controls regularly made it beyond 90% of the field (Figure 5C). Even so, the wave in AxD astrocytes propagated at ~5 μm/s for both mutations, significantly slower than 10–15 μm/s for all control cells (Figure 5D), despite similar number of cells per field as examined by post-experiment cell counts (Figure 5E). Thus, human AxD astrocytes are capable of releasing intracellular calcium stores but are defective in propagating calcium waves from one cell to another.

To determine if astrocytes in AxD transgenic mice exhibit altered calcium signaling, we isolated primary astrocytes from neonatal R236H/+ transgenic mice (Hagemann et al., 2006; Kong et al., 2016) and cultured them under the same conditions as the human pluripotent stem cell (hPSC)-derived astrocytes. The average rate of calcium wave propagation in wild-type mouse astrocytes was ~8 μm/s (Figures S6D and S6E) for both lines. The AxD mouse astrocytes demonstrated about a 50% reduction in rate of calcium wave propagation, averaging ~4 μm/s in both lines used (Figures S6D and S6E). The wild-type rates are similar to reported rodent calcium wave kinetics (Haas et al., 2006; Oberheim et al., 2009), and the reduction in AxD mouse astrocytes matches what we observed in the human astrocytes. Interestingly, though, despite a reduced rate of calcium wave propagation, the AxD mouse astrocytes rarely failed to propagate across the entire field of view with both wild-type and AxD astrocytes propagating across the field similarly (Figure S6F), differentiating them from the human astrocytes. Thus, calcium wave propagation is attenuated in AxD mice.

### AxD Astrocytes Produce and Sense but Fail to Release ATP

**Figure 6.**
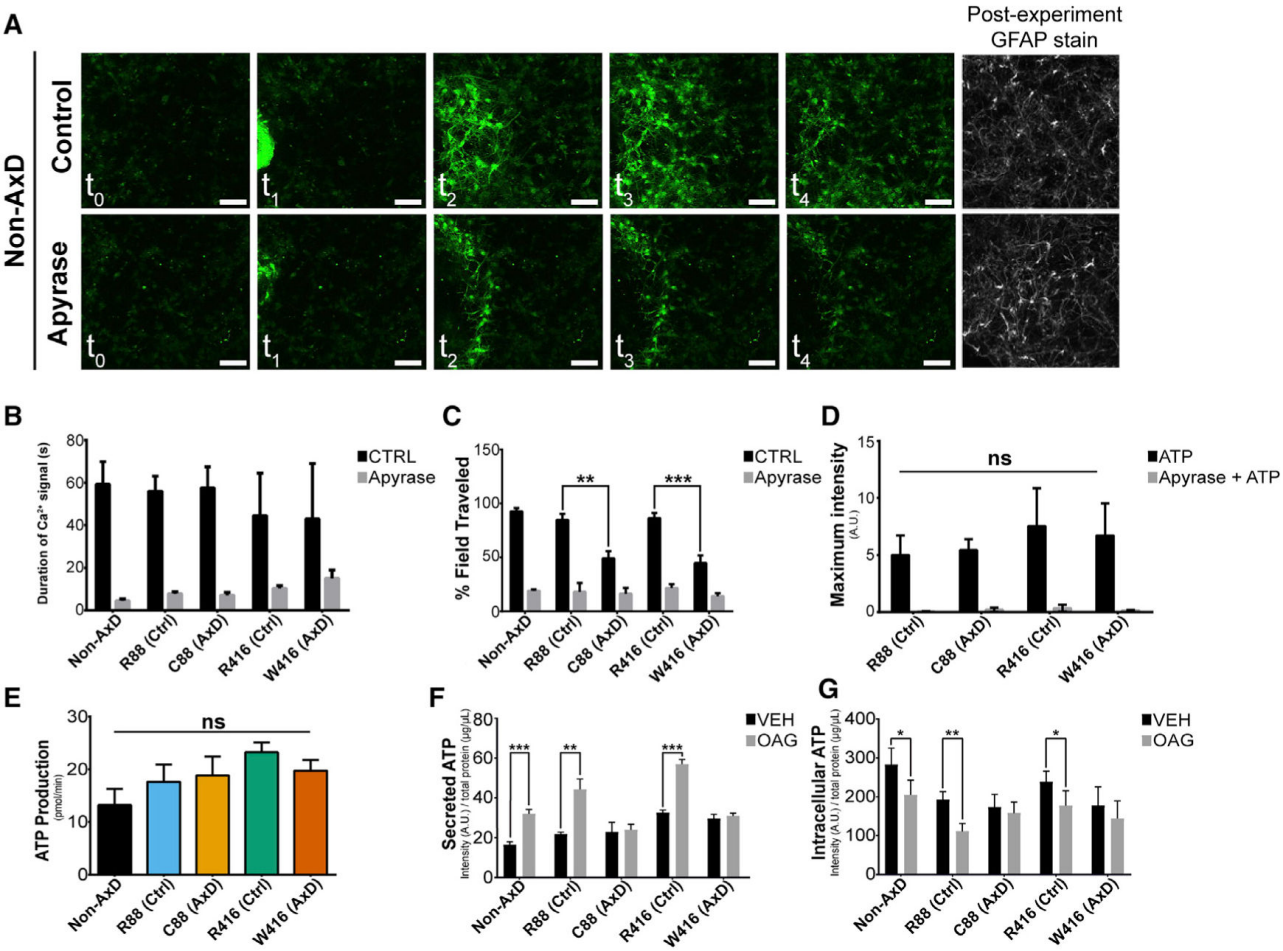
ATP Secretion Deficits in AxD Astrocytes (A) Still images over the duration (2 min) of mechanical stimulation on wild-type 6 month astrocytes ± apyrase with accompanying post-experiment GFAP stains. (B) Average duration of increased calcium signal above threshold ± apyrase (n = 10). (C) Average percentage of field traveled by Ca^2+^ wave ± apyrase (n = 10). (D) Maximum fluorescence intensity achieved after bath application of 100 μm ATP ± apyrase (n = 10). (E) Average results of ATP production via seahorse metabolic profiling (n = 3). (F) Average ATP concentration detected in media ± OAG treatment (n = 3). (G) Average ATP concentration remaining in cells ± OAG treatment (n = 3). **p = 0.001–0.01 and ***p < 0.001. Scale bars, 100 μm. Data are represented as ± SEM.

Calcium wave propagation is mediated, in part, by extracellular ATP. The reduced calcium wave propagation in AxD astrocytes may be attributed to failure in production, release, and/or detection of ATP. As mitochondria and metabolic pathways were implicated by RNA sequencing (RNA-seq), we profiled the metabolism of astrocytes from all groups via Seahorse metabolic flux analysis. Interestingly, we observed a marked reduction in extracellular acidification resulting from glycolysis, which was commensurate with an increased electron transport chain activity in both disease lines, but no significant difference in ATP production was observed (Figures 6E and S5). We also observed that AxD astrocytes were operating near their theoretical maximal cellular respiration (Figure S5F), likely compensating for reductions in glycolysis. These results suggest that though AxD astrocytes have altered metabolic profiles, they are able to produce ATP at a rate similar to that of control lines but may have a limited capacity to further increase ATP production.

We then asked if AxD astrocytes respond to ATP. When astrocytes were mechanically stimulated in the presence of the ATP degrading enzyme apyrase, calcium waves were completely ablated in all groups (Figures 6A–6C). Bath application of ATP to fluo-4-loaded astrocytes stimulated robust increases in fluorescence intensity across all groups, which was prevented in the presence of apyrase or the competitive inhibitor oATP (Figures 6D and S6A). These results suggest that the purinergic signaling is intact in AxD astrocytes.

The fact that AxD astrocytes produce and respond to ATP suggests that failed ATP release is the main mechanism behind the AxD defect in ATP-mediated calcium wave propagation. We therefore directly measured intracellular and secreted ATP in the absence or presence of 1-oleoyl-2-acetyl-sn-glycerol (OAG), an analog of diacylglycerol that mobilizes intracellular calcium stores and stimulates extracellular ATP release (Mungenast, 2011). Upon OAG stimulation, the levels of extracellular ATP were significantly increased in the media of control but not AxD astrocyte cultures (Figure 6F). Intracellular ATP levels were commensurately reduced in control lines but unchanged in AxD lines (Figure 6G). This effect was lost when OAG was administered in the presence of the IP3R inhibitor 2-APB (Figures S6B and S6C). Importantly, total ATP levels were not different between any of the groups, demonstrating that indeed ATP release is defective in AxD astrocytes.

## DISCUSSION

Using the iPSC model, we found that AxD astrocytes present GFAP aggregation, recapitulating the core pathology in AxD patients and transgenic animals. Unbiased RNA-seq revealed changes in pathways that broadly implicate the ER, vesicle regulation, and cellular metabolism. This is confirmed by morphological alteration and perinuclear localization of ER and lysosomes. Importantly, we found that AxD astrocytes fail to propagate calcium waves across astrocytes, and this deficit is attributed to the impaired ATP release from astrocytes. These morphological and functional phenotypes were prevented by correction of GFAP mutations, highlighting the role of mutant GFAP in causing disease-relevant phenotypes. Our findings reveal unappreciated roles of GFAP associated with ER and lysosomes, vesicle regulation, and secretion.

AxD is caused by mutations in the GFAP gene, and its pathological hallmark is RF formation. Our AxD iPSC-derived astrocytes exhibit RF-like GFAP inclusions as they contain GFAP, αB-crystallin, and vimentin, which is typically seen in AxD patients and transgenic animals. Morphologically, the RF-like aggregates observed in our model are less dense and are primarily localized to the perinuclear area. We observed RF formation in our AxD astrocytes without obvious increases in overall GFAP protein levels, though interestingly, disease astrocytes exhibited lower GFAP transcripts in our RNA-seq data. This may appear to contradict the RF formation with higher levels of GFAP seen in AxD patients and transgenic animals. However, upregulations in GFAP are often associated with gliosis in AxD patients and animal models (Hagemann et al., 2005; Tang et al., 2008). GFAP aggregation without an increase in GFAP expression may represent early nascent RFs. It suggests that mutations in GFAP are sufficient to induce aggregation. This phenomenon enables us to investigate the early primary impacts of mutated GFAP protein, separating from downstream effects secondary to disease progression.

How mutant GFAP results in astrocyte dysfunction and global pathological changes in AxD remains elusive. AxD transgenic animals resemble AxD by presenting RFs and demonstrating an increased stress response and susceptibility to kainic acid-induced seizures (Hagemann et al., 2006). However, the exact cellular mechanism remains unknown. The relatively mild phenotypes in AxD transgenic animals compared with human patients make it difficult to dissect the cellular and molecular mechanisms of AxD pathogenesis. Recently, Kondo et al. (2016) found upregulation in several cytokines and increased mTOR activation in AxD patient iPSC-derived astrocytes. This is similar to the observations in transgenic animals when gliosis is obvious (Hagemann et al., 2005; Pekny et al., 2014; Tang et al., 2008). However, our unbiased RNA-seq analysis using isogenic cells points to the association between GFAP mutations and altered vesicular regulation. Indeed, AxD astrocytes show swollen ER and lysosome clustering, restricted to a perinuclear area. Such morphological alterations suggest disruption of endo- and exocytotic pathways, altered lipid biosynthesis and/or vesicular formation. Disruptions in membrane compartments and membrane biosynthesis have been associated with other intermediate filaments (Schweitzer and Evans, 1998; Styers et al., 2005). Vimentin directly interacts with the Golgi apparatus (Gao and Sztul, 2001) and is involved in membranous protein trafficking (Gillard et al., 1998; Sarria et al., 1992). Mice lacking vimentin and GFAP have stimulation-dependent endosome and exosome (lysosome) trafficking deficits (Potokar et al., 2010). Hence, it is not strange that GFAP is involved in similar processes given its intermediate filament nature.

Astrocytes communicate to one another through calcium signaling, which is in turn mediated by regulated release of ATP (Bazargani and Attwell, 2016; Verkhratsky and Nedergaard, 2018). We therefore focused on the biogenesis and trafficking of ATP to understand the role of mutant GFAP on membrane biogenesis and trafficking. Our systematic analysis revealed that AxD astrocytes synthesize and respond to, but fail to release, ATP. Because extracellular apyrase significantly attenuated calcium wave propagation, we concluded that gap junctions were likely not the main mechanism behind our observations. Our data suggest that it is the extracellular release of ATP from astrocytes, likely lysosomal, that is altered in AxD astrocytes. The enlarged lysosomes with restricted localization to the perinuclear compartment may interfere with their fusion with cell membrane for ATP release. The reduced level of extracellular ATP may be further aggravated by altered uptake. The consequence of failed ATP release is disruption of astrocyte-astrocyte communication, a critical role of astrocytes. This would also interrupt the communication between astrocytes and neurons as well as other glial cells. These results help explain why GFAP mutations in astrocytes lead to global neurological deficits in the CNS of AxD patients.

The analysis of the cellular outcomes of mutant GFAP has led to the realization of fundamental biological roles for GFAP. To date, few non-structural functions have been assigned to GFAP, and none of the available AxD animal models have demonstrated the functional changes we have observed in human patient-derived cells. The disrupted distribution of ER and lysosomes in the presence of GFAP mutations strongly suggests that GFAP, like other intermediate filaments, plays a pivotal role in vesicle regulation, specifically lysosome-mediated exocytosis of ATP. Further analysis of the AxD iPSC model will likely reveal more detailed mechanistic roles for GFAP.

## CONTACT INFORMATION

Further information and requests for resources and reagents should be directed to and will be fulfilled by the lead contact, Su-Chun Zhang (Zhang@waisman.wisc.edu)

## CELL LINE INFORMATION

### iPSC generation

Dermal fibroblasts were obtained by the Waisman center iPSC core (http://www.waisman.wisc.edu/cores-idd-ipsc-models.htm), with oversight from the institutional review board (IRB). Patient WC-01-01-AL-AM was a 5yo male heterozygous for R88C GFAP mutation. Patient WC-14-01-AL-AM was an 8yo male heterozygous for R416W GFAP mutation. Fibroblasts were tested for mycoplasma and expanded. Cells were virally reprogrammed with Yamanaka factors and observed for morphology changes. Clones were picked, sub-cloned, and validated for pluripotency by immunofluorescent staining.

### Mouse Primary Astrocytes

R236H/+ neonatal pups (Hagemann et al., 2006) were generously provided by Dr. Albee Messing. Primary astrocytes were isolated with the approval by the Institutional Animal Care and Use Committee (IACUC) at the University of Wisconsin-Madison, using previously described techniques (Kong et al., 2016).

## METHOD DETAILS

### CRISPR/CAS9 corrections

PAMsites were identified 200bp up and downstream of affected exon and guide RNAs were designed via MIT CRISPR Design (http://crispr.mit.edu). Wild-type donor exons were cloned out of a non-AxD cell line (human embryonic stem cell line, H1) and inserted into a donor vector designed with 1kb homology arms. The donor vector contained a floxed geneticin selection cassette which was designed to integrate at least 200bp upstream or downstream of affected exon in the intron. Positive clones were verified by PCR and sub cloned twice while under selection to ensure clonality. Two sub clones for each mutant and corrected line were validated and maintained for use throughout this study.

### Cell culture and differentiation

All cells were cultured at 37°C with an atmosphere maintained at 5% O_2_ and 5% CO_2_. iPSCs were maintained on matrigel in TeSR-E8 media. Cells were passaged every 6-7 days in the presence of ROCK inhibitor to promote cell survival. Neural induction was mostly performed via monolayer dual SMAD inhibition (SB431542; DMH1) (Chambers et al., 2009). Neuroepithelia in the rosettes were lifted 15 days after the start of neural induction and propagated to generate astrocytes as previously described (Krencik et al., 2011). 6-month astrocyte progenitor cells were enzymatically digested with Trypsin (GIBCO) and plated as single cells for maturation. Maturation media was composed of DMEM/F12 containing 1x N2, 1x NEAA, 1x Glutamax, 1x pen-strep and supplemented fresh with 10ng/mL BMP4 and 10ng/mL CNTF. Media was fully changed every other day for 1 week before experiments.

### Calcium wave experiments

Astrocytes were plated on Cellvis 35mm glass bottom dishes at 60,000 cells per dish. Cells were matured as described above. Fluor4-AM was applied to cells at a final concentration of 5 μM along with pluronic F-127 (1:1000) for 30 minutes prior to use. Cells were then washed once with DPBS (1x) and switched to 2mL of pre-warmed phenol free neurobasal before experiments. A flame-polished Pasteur pipette was prepared in advance and painted with matte black nail-polish to reduce laser scattering. A manual micromanipulator was mounted on a custom-built stage which allowed the Pasteur pipette to reach the plated cells. All imaging was performed on a Nikon A1 confocal using resonant scanning at maximum frames per second (24FPS) in Nikon Elements software. All samples were recorded for 2 minutes post stimulation.

### Dextran Endocytosis Assay

Astrocytes were plated into Perkin-Elmer 96-well imageing plates at 10,000 cells per well. pHrodo green dextran and Cell Mask Orange were applied to cells per manufactures instructions. Astrocytes were cultured for 8 hours in a Perkin Elmer Operetta under standard cell culture conditions. Images were taken every 30 minutes at 20x. Analysis was performed by identifying cells via cell mask orange. Green punctate were considered endocytosed if they appeared inside the cell boundaries defined by cell mask orange.

### ATP Release Assay

1-Oleoyl-2-acetyl-sn-glycerol (OAG) was purchased from Caymen Chemical and prepared at 10mg/ml (25mM) in DMSO under pure nitrogen and used fresh or flash frozen and stored at −80°C. OAG was used at a final concentration of 100 μM for 15 minutes. Astrocytes were plated in a 96-well dish at a density of 12,000 cells per well and matured prior to experiments. Before starting the experiment, cells were switched to HBSS in a minimal volume and allowed to equilibrate in the incubator for 30min. Treatments were then added by multichannel pipette and strictly timed. After 15minutes, media was removed from cells and ATPassay was performed per manufacturer’s instructions. Immediately following removal, cell lysis buffer was added to the wells and the ATPassay was performed in intracellular content via manufacturer’s instructions.

### ATP Stimulation

Astrocytes were plated on Cellvis 35mm glass bottom dishes at 30,000 cells per dish and matured as described above. Fluor4-AM was applied to cells at a final concentration of 5 mM along with pluronic F-127 (1:1000) for 30 minutes prior to use. Cells were then washed once with DPBS (1x) and switched to 2mL of pre-warmed phenol free neurobasal containing vehicle or inhibitors. Imaging was performed on a Nikon A1 confocal using resonant scanning at maximum frames per second (24FPS) in Nikon Elements software. A baseline was recorded for 10-15 s and then ATP was added dropwise at the edge of the coverslip to a final concentration of 100 or 10μM.

### Immunocytochemstry

Cells were fixed in 4% paraformaldehyde for 20 minutes at room temperature. CNX43 stains required fixation with ice cold methanol for 20 minutes. Cells were blocked and permeabilized during a single 1 hour incubation at room temperature with 4% donkey serum and 0.1% Triton X-100.

### RNA-seq

Astrocyte groups were split into different flasks at 4months of culture and expanded as spheres for 2 months. At 6 months of culture, astrospheres were broken to single cells via trypsin and plated in triplicate onto matrigel coated 6-well plates at 300,000 cells per well. Astrocytes were matured following the protocol described above. RNA was collected using RNeasy plus mini kit and 260/280 ratios were all approximately 2. ~2μg RNA per replicate was submitted to the UW-Madison biotechnology center where RNA quality was assessed using an Agilent RNA PicoChip. Sample libraries were prepared using poly-A selection using an Illumina TruSeq RNAv2 kit following manufacturer’s instructions.

Prepared libraries were sequenced for 101-bp single-read and performed on an Illumina HiSeq 2500 using 1X100 sequencing to a read depth of >25 million reads per sample by the University of Wisconsin-Madison DNA Sequencing Facility in the University of Wisconsin-Madison Biotechnology Center. FastQC (RRID: SCR_014583) was performed on all samples with every sample passing all quality control measurements.

### Quantitative PCR

RNA was extracted from 6-month progenitor cells by the QIAGEN RNeasy Plus Mini Kit and quantified. Equal amounts of RNA were used for reverse transcription, performed according to manufacturer specifications, using Bio-Rad iScript. iTaq universal SYBR Green Supermix was used for all reactions. Housekeeping gene assays were performed and several candidate genes were identified. For all experiments, FTH1 and Actin were was used for internal normalization.

### Seahorse Metabolic Assays

For all assays, astrocytes were plated in a Seahorse assay plate at a density of 5,000 cells per well and matured for 1 week prior to testing. All media and kits were purchased from Seahorse biosciences and dose responses were characterized before experiments. Cells were analyzed in a Seahorse Metabolic Flux Analyzer XF96. For post-assay, cells were fixed in 4% PFA and quantification was performed via Cresyl violet absorbance assay.

#### Glycolysis stress test

Astrocytes were washed and switched to unbuffered Seahorse media devoid of glucose and allowed to equilibrate for 1 hour. A series of pH measurements were taken before injection of 10 μM glucose to assess baseline ECAR. 1 molecule of glucose can yield up to 2 molecules of lactic acid and 2 free protons which are shuttled out of the cell, contributing to an increase in extracellular pH (Mookerjee et al., 2015). However, the full utilization of glucose all the way through the tricarboxylic acid cycle (TCA) yields 6 molecules of carbonic acid (from CO^2^) and 6 free protons, thus confounding ECAR by contributing more acidic molecules to the extracellular environment. We therefore injected 1 μM of Oligomycin A, an ATPase inhibitor, to ablate the generation of acidic molecules from the oxidation of glucose. Finally, 1 μM of 2-Deoxyglucose, a competitive inhibitor of the rate limiting glycolytic enzyme hexokinase, was injected to stop the experiment.

#### Mitochondrial Stress Test

Cells were cultured in unbuffered cell culture medium containing 10 μM glucose. Baseline measurements were taken under these conditions before a series of molecules were injected into the medium to halt oxidative phosphorylation at different steps. First, 1 μM Oligomycin was injected to halt all ATP synthesis, allowing measurement of ATP production rate. Next, the electron transport chain decoupling agent, FCCP was injected at a final concentration of 1 μM, allowing analysis of maximal respiration rate. Finally, a combination of Antimycin A and Rotenone were injected at a concentration of 0.5 μM each, to inhibit the electron transport chain and yield the spare respiratory capacity of the astrocytes.

Statistics were performed in a pairwise fashion, utilizing unpaired Student’s t test to compare each disease line to its respective corrected control in Graphpad Prism. * = p ≤ 0.05, ** = p 0.001 to 0.01, *** = p 0.0001 to 0.001

## QUANTIFICATION AND STATISTICAL ANALYSES

### ATP Stimulation analysis

Raw video files were loaded into Nikon Elements Analysis software and the entire field was selected as the region of interest (ROI). Intensity was measured for every frame of the video. The first 20-30 frames were averaged together as a baseline intensity and compared against the peak intensity value. Statistics were performed in a pairwise fashion, utilizing unpaired Student’s t test to compare each disease line to its respective corrected control in Graphpad Prism. * = p ≤ 0.05, ** = p 0.001 to 0.01, *** = p 0.0001 to 0.001

### Calcium wave analysis

Raw video files were loaded into Nikon Elements Analysis software. The first frame of increased fluorescent intensity was manually identified and recorded. The total potential distance was identified by measuring the distance form the leading edge of increased signal to the opposite side of the field. The final frame of the calcium event was determined either by the wave spreading across the entire field or when the last pixel returned to a baseline intensity the time of this event was used for the total time of calcium event. Statistics were performed in a pairwise fashion, utilizing unpaired Student’s t test to compare each disease line to its respective corrected control in Graphpad Prism. * = p ≤ 0.05, ** = p 0.001 to 0.01, *** = p 0.0001 to 0.001

### Immunocytochemical Analysis

Quantification was performed on ImageJ software including FIJI plug-in packages. In brief, all images were background subtracted and thresholds were set by the software. Processes were defined as any structure coming off the main cell body with a width of at least 2 μm. These regions were then manually outlined as ROIs and intensities were measured. Nuclei were manually counted using the multi-point tool. For all experiments, at least three coverslips were analyzed per group. Statistics were performed in a pairwise fashion, utilizing unpaired Student’s t test to compare each disease line to its respective corrected control in Graphpad Prism.* = p ≤ 0.05, ** = p 0.001 to 0.01, *** = p 0.0001 to 0.001

### RNA-seq and Pathway analysis

#### EBSeq

The empirical Bayes hierarchical modeling approach EBSeq (RRID: SCR_003526) was used to identify differentially expressed genes (DEGs) between disease and corrected groups. Median normalization technique of DESeq (RRID: SCR_000154) (Anders and Huber, 2010) was used to account for differences in sequencing depth. EBSeq calculates the posterior probability (PP) of a gene being in each expression pattern. Genes were declared differentially expressed at a false discovery rate controlled at 100*(1- α) % if the posterior probability of P1 (EE) is less than 1- α. Given this list of DE genes, the genes are further classified into each pattern and sorted by PP.

#### Clustering, pathway and gene ontology analysis

Hierarchical clustering was achieved via Morpheus (https://software.broadinstitute.org/morpheus). In general, we found Enrichr (RRID: SCR_001575) (http://amp.pharm.mssm.edu/Enrichr/) (Chen et al., 2013; Kuleshov et al., 2016) to be most useful for pathway and gene ontology analysis.

EBSeq DEGs from each group were analyzed for differentially regulated pathways using Enrichr which utilizes several pathway databases for general pathway analysis.

Additional DEGs were identified between disease and correction by averaging normalized TPM values of replicates, and removing any genes with a TPM < 100. Ratios of these values (disease and corrected) were then compared against each other and only genes which changed by at least ≥ 2-fold were analyzed. These values were also used in Enrichr to identify pathways and gene ontologies which were upregulated and downregulated.

R-script for highest variable (over-dispersed) gene expression was followed as previously described (Fan et al., 2016; Sloan et al., 2017).

## Supplementary Material

1

2

3

4

5

## Figures and Tables

**Table:** KEY RESOURCES TABLE

REAGENT or RESOURCE	SOURCE	IDENTIFIER
Antibodies		
Mouse anti-αB Crystallin	Abcam	Cat# ab13496, RRID: AB_300400
Rabbit anti-Connexin43	Abcam	Cat# ab11370, RRID: AB_297976
Rabbit anti-ERp57	Proteintech	Cat# 15967-1-AP, RRID: AB_2236784
Rabbit anti-GFAP	DAKO	Cat# Z0334, RRID: AB_10013382
Mouse anti-GFAP	Millipore	Cat# IF03L, RRID: AB_2294571
Rabbit anti-GM130	Cell Signaling Technologies	Cat# 2296S, RRID: AB_10695240
Mouse anti-LAMP2	Novus	Cat# NBP2-22217, RRID: AB_2722697
Mouse anti-S100B	Abcam	Cat# ab66028, RRID: AB_1142710
Goat anti-Sox9	R&D Systems	Cat# AF3075, RRID: AB_2194160
Rat anti-tubulin	Abcam	Cat# ab6160, RRID: AB_305328
Alexa Fluor 546 donkey anti-rabbit IgG (H+L)	Molecular Probes	Cat# A10040
Alexa Fluor 488 donkey anti-mouse IgG (H+L)	Molecular Probes	Cat# A21202
Alexa Fluor 488 donkey anti-goat IgG (H+L)	Molecular Probes	Cat# A11055
Alexa Fluor 647 Donkey Anti-rat IgG (H+L)	Abcam	Cat# ab150155
Alexa Fluor 647 Donkey Anti-mouse IgG (H+L)	Molecular Probes	Cat# A-31571
Critical Commercial Assays		
ATPlite Luminescence Assay	Perkin-Elmer	Cat# 6016941
RNeasy mini kit	QIAGEN	Cat# 74106
Seahorse XF Cell Mito Stress Test Kit	Seahorse Biosciences	Cat# 103015-100
Seahorse XF Glycolytic Rate Assay Kit	Seahorse Biosciences	Cat# 103344-100
Experimental Models: Cell Lines		
H1 human embryonic stem cells	WiCell Research Institute	WA01
AxD patient fibroblasts (C88)	Waisman center iPSC core	WC-01-01-AL-AM
AxD patient fibroblasts (W416)	Waisman center iPSC core	WC-14-01-AL-AM
R236H/+ primary astrocytes	Generously provided by Dr. Albee Messing	N/A
Software and Algorithms		
Enrichr	Ma’ayan lab	http://amp.pharm.mssm.edu/Enrichr/
Harmony	PerkinElmer	http://www.perkinelmer.com/product/harmony-4-6-office-hh17000001
ImageJ	National Institutes of Health	https://imagej.nih.gov/ij/
Morpheus	Broad Institute	https://software.broadinstitute.org/morpheus/
NIS Elements	Nikon	https://www.nikoninstruments.com/Products/Software
Prism	GraphPad	https://www.graphpad.com/scientific-software/prism/
R-Studio	RStudio	https://www.rstudio.com

## Highlights

- GFAP mutations result in mislocalization of organelles
- AxD astrocytes display impaired ATP release
- AxD astrocytes fail to propagate calcium waves
- Correction of GFAP mutations restores organelle distribution and astrocyte function
