# Supplementary material for: Mutations in GFAP Disrupt the Distribution and Function of Organelles in Human Astrocytes
Source: Cell Rep. Author manuscript; Available in PMC 2018 Dec 2. (PMC6275075; doi:10.1016/j.celrep.2018.09.083)
Supplement: 1 [file NIHMS1511534-supplement-1.pdf]

Cell Reports, Volume *25*

Supplemental Information

Mutations in GFAP Disrupt the Distribution and Function of Organelles in Human Astrocytes

Jeffrey R. Jones, Linghai Kong, Michael G. Hanna IV, Brianna Hoffman, Robert Krencik, Robert Bradley, Tracy Hagemann, Jeea Choi, Matthew Doers, Marina Dubovis, Mohammad Amin Sherafat, Anita Bhattacharyya, Christina Kendziorski, Anjon Audhya, Albee Messing, and Su-Chun Zhang

Supplemental Information

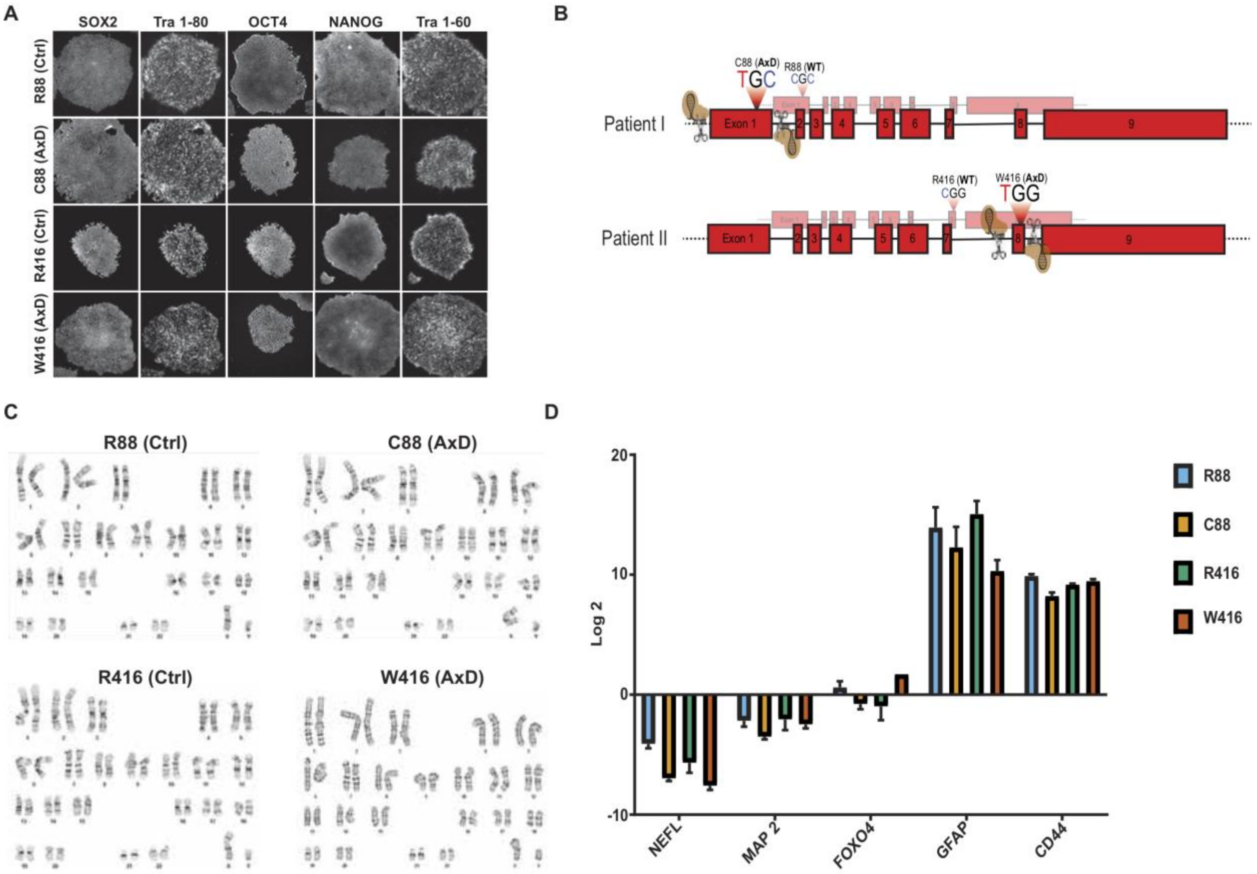

Figure S1. Cell line characterization, Related to Figure 1.
(A) Panel of immunostaining on iPSC colonies from all groups. Pluripotency markers SOX2, Tra1-80, OCT4, NANOG, & Tra-160 were probed.
(B) Cartoon depicting gene editing strategy. The entire mutant exon was replaced with a donor wild-type exon.
(C) Representative karyotyping results from each iPSC line after 40 passages.
(D) Quantitative PCR on 6-month astrocytes from each group and hPSC derived neurons. Log2 expression values are shown for astrocyte samples relative to neuronal values for each gene. n=3. Error bars are mean ± SD

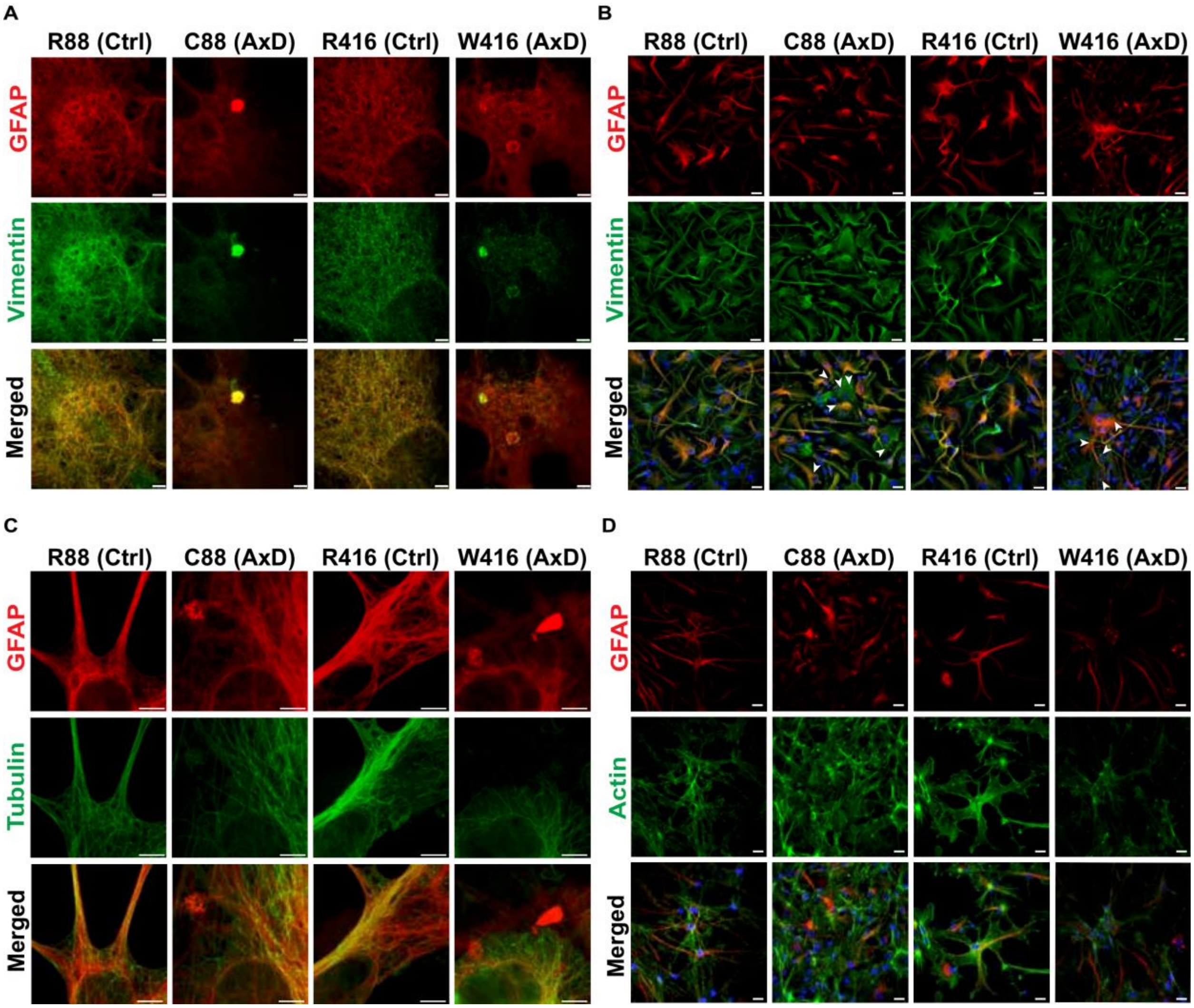

Figure S2. GFAP inclusions co-localize with vimentin, but no obvious change in other cytoskeletal polymerization, related to Figures 2 & 4.

(A) Super resolution STED images of single astrocytes from each group at 6-months. Filamentous GFAP (red) and vimentin (green) were evident with co-localization. Scale bars = 10μm.

(B) Panel of immunostaining on 6-month astrocytes. Astrocytes were immunopositive for intermediate filament proteins GFAP (red) and vimentin (green). Punctate GFAP and vimentin co-localized (white arrow heads). Scale bars = 50μm.

(C) Super resolution STED images of single astrocytes from each group at 6-months. Filamentous GFAP (red) and tubulin (green) were evident. Scale bars = 10μm.

(D) Immunofluoresent images of 6-month astrocytes probed for GFAP (red) and filamentous actin (green). Scale bars = 50μm.

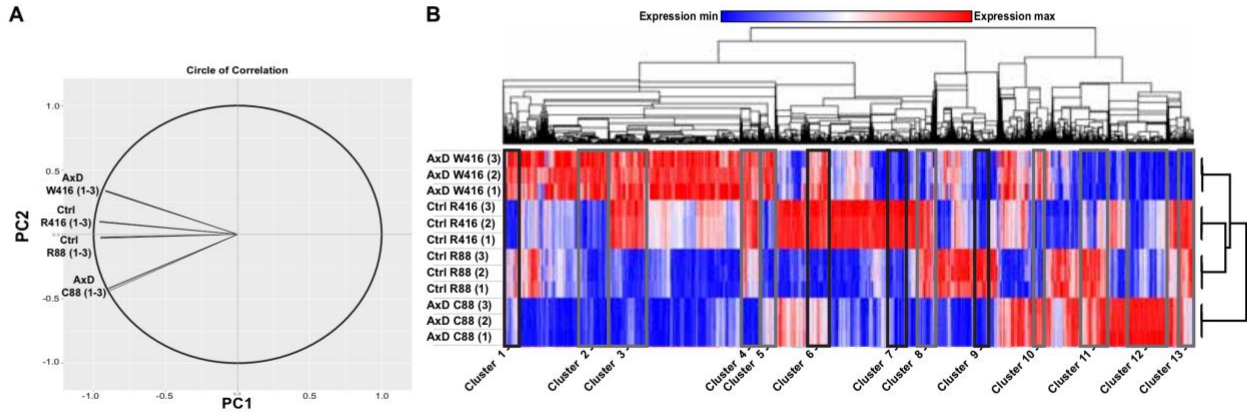

C

Cluster 1: R416 (Ctrl) Unique Down GO Terms	P-value	Cluster 6: R88 (Ctrl) Unique Down GO Terms	P-value	Cluster 7: R416 (Ctrl) Unique Up GO Terms	P-value	Cluster 9: R88 (Ctrl) Unique Up GO Terms	P-value
regulation of transcription from RNA polymerase II promoter	3.5E-10	retrograde transport, endosome to Golgi	7.7E-6	MHC class II protein complex binding	6.2E-7	Cell cycle	2.2E-4
negative regulation of transcription, DNA-templated	1.3E-8	ubiquitin-dependent protein catabolic process	2.1E-5	MHC class II receptor activity	1.0E-6	Neuroactive ligand-receptor interaction	1.7E-3
regulation of DNA-templated transcription, initiation	2.0E-7	regulation of multivesicular body size	2.5E-5	MHC class II protein binding, via antigen binding groove	2.8E-6	Homologous recombination	2.7E-3
chromatin organization involved in regulation of transcription	5.1E-7	neurotransmitter receptor transport, plasma membrane to endosome	3.4E-4	exo-alpha-(2->3)-sialidase activity	1.9E-4	Glutamatergic synapse	8.7E-3
regulation of antisense RNA transcription	5.1E-7	ubiquitin-dependent SMAD protein catabolic process	4.6E-4	exo-alpha-(2->6)-sialidase activity	1.9E-4	Glycerophospholipid metabolism	1.3E-2

Figure S3. Transcriptome profiling of control lines, Related to Figure 3.

(A) Circle of correlation obtained from principle component analysis of full transcriptomic data set.

(B) Hierarchical clustering heatmap of full transcript data set.

(C) Table of the most significant gene ontology terms specific to corrected line clusters.

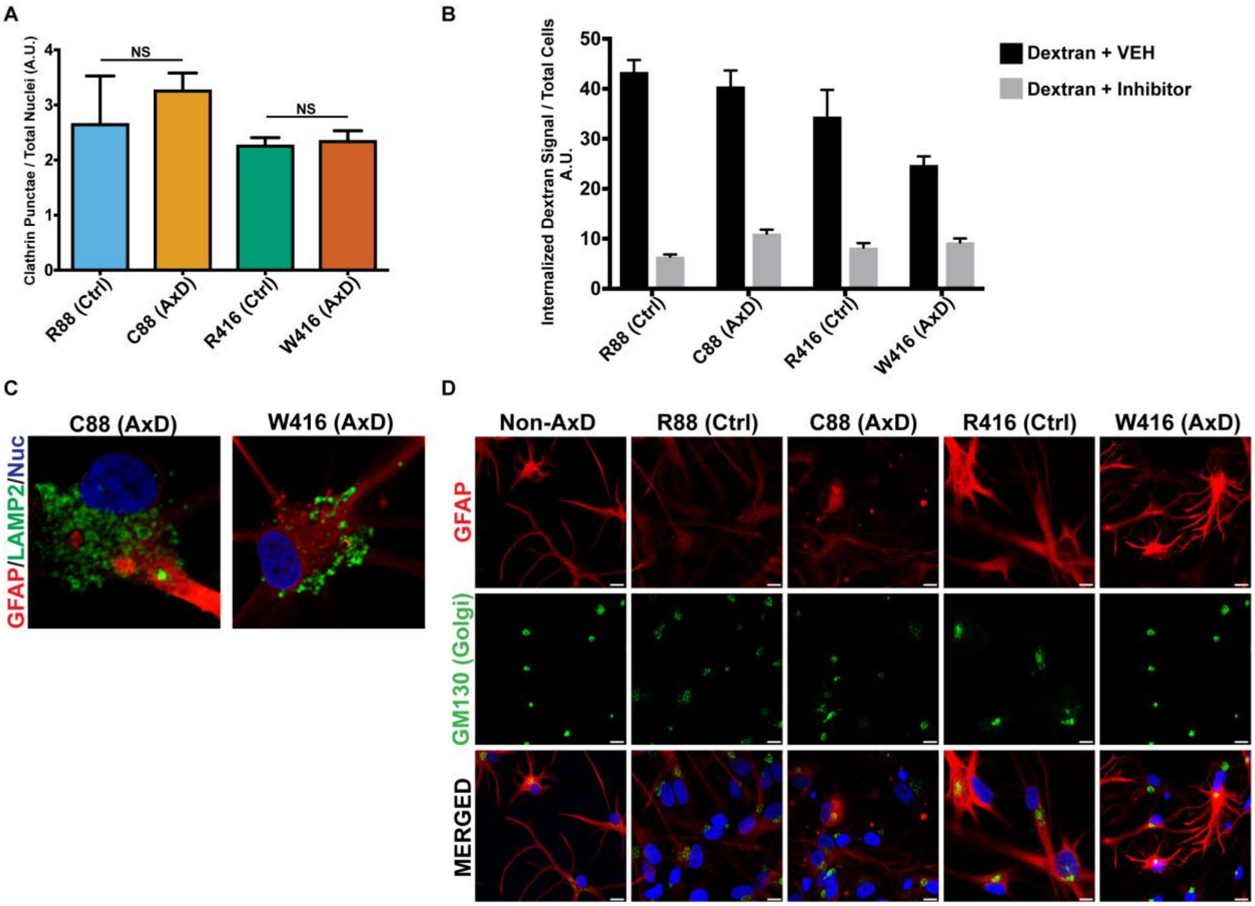

Figure S4. Mutant GFAP does not alter basal endocytosis and RFs are not localized to lysosomes, Related to Figure 3 & 4.

(A) Results of high-content imagining of clathrin puncta within 6-month astrocytes. n=3.

(B) Quantification of high-content imaging of live 6-month astrocytes incubated with fluorescent dextran ± Brefeldin A (5µg/ml). n=4.

(C) Immunocytochemistry for GFAP (red) aggregates and LAMP vesicles (green) demonstrating no co-localization.

(D) Immunocytochemistry on 6-month astrocytes, probed for GFAP (red) and Golgi marker GM130 (green). Scale bars = 50µm. NS = not significant. Error bars are mean ± SD.

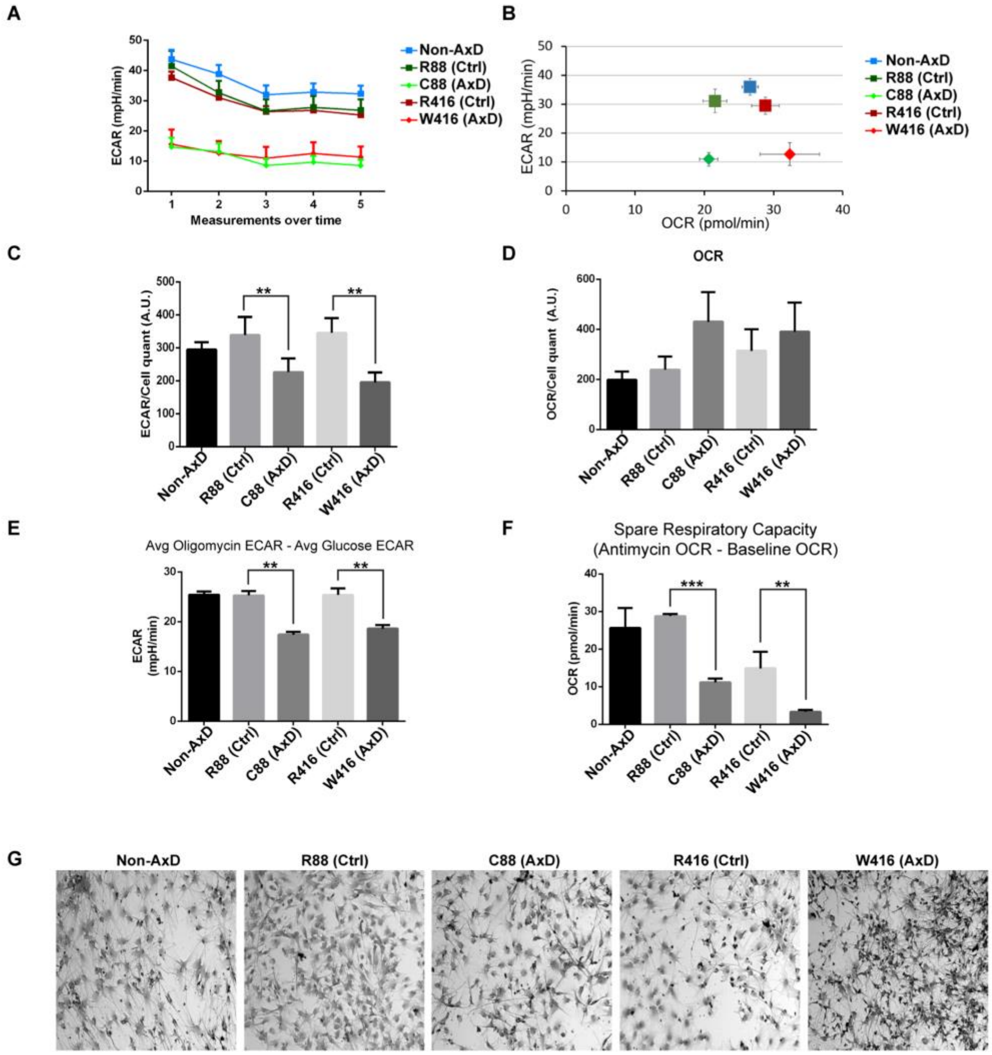

Figure S5. Metabolic profiling of astrocytes, Related to Figure 5 & 6.
(A) Graph of extracellular acidification rates (ECAR; pH change) over time for 6-month astrocytes. n=3.
(B) Graph comparing ECAR versus oxygen consumption rates (OCR) for all groups at 6 months.
(C) ECAR rates normalized to post-experiment cell quantification. n=3.
(D) OCR rates normalized to post-experiment cell quantification. n=3.
(E) Quantification of glycolysis rates on 6-month astrocytes using Seahorse glycolysis stress test. Bars represent the average value of ECAR after oligomycin injection minus average ECAR from glucose injection. n=3.
(F) Quantification of spare respiratory capacity of the electron transport chain on 6-month astrocytes using Seahorse mitochondrial stress test. Bars represent the average OCR after antimycin injection minus the average OCR from baseline. n=3.
(G) Representative plating densities of astrocytes, fixed post experiment and stained with Cresyl Violet. Error bars are mean ± SD. * = $p \leq 0.05$, ** = $p \leq 0.01$

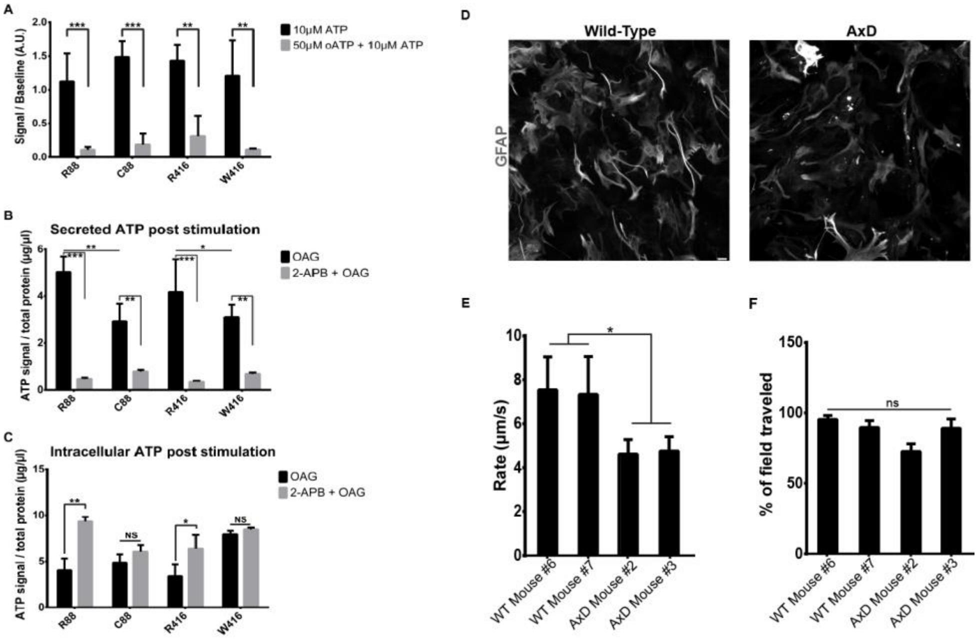

Figure S6. Inhibition of intracellular Ca^{2+} release via 2-APB on human astrocytes and Ca^{2+} wave analysis in primary astrocytes from AxD mice, Related to Figures 5 & 6.

(A) Quantification of fluorescent intensity increase over baseline in 6-month astrocytes loaded with Fluor4-AM and treated with 10μm ATP ± the competitive inhibitor oATP. n=4.

(B) Extracellular ATP measured in the media from 6-month astrocytes after treatment with OAG ± 2-APB. n=4.

(C) Intracellular ATP levels quantified after secretion in 6-month astrocytes after treatment with OAG ± 2-APB. n=4.

(D) Immunocytochemistry for GFAP on primary astrocytes from AxD mice (R236H/+) or WT littermate.

(E) Rate of Ca^{2+} wave propagation across astrocytes derived from 4 mice. n=3.

(F) Average percentage of field traveled by Ca^{2+} wave. n=3.

A-C are represented as mean ± SD. E & F are represented as mean ± SEM.

ns = not significant. *$p \leq 0.05$, **$p = 0.001$ to 0.01, ***$p < 0.001$.
